# Toll-like receptor polymorphisms in malaria-endemic populations

**DOI:** 10.1186/1475-2875-8-50

**Published:** 2009-03-24

**Authors:** Jennifer A Greene, Ann M Moormann, John Vulule, Moses J Bockarie, Peter A Zimmerman, James W Kazura

**Affiliations:** 1 Center for Global Health and Diseases, Case Western Reserve University, Cleveland OH, USA; 2 Kenya Medical Research Institute, Kisumu, Kenya; 3 Papua New Guinea Institute of Medical Research, Madang, Papua New Guinea

## Abstract

**Background:**

Toll-like receptors (TLR) and related downstream signaling pathways of innate immunity have been implicated in the pathogenesis of *Plasmodium falciparum *malaria. Because of their potential role in malaria pathogenesis, polymorphisms in these genes may be under selective pressure in populations where this infectious disease is endemic.

**Methods:**

A post-PCR Ligation Detection Reaction-Fluorescent Microsphere Assay (LDR-FMA) was developed to determine the frequencies of *TLR2, TLR4, TLR9*, *MyD88-Adaptor Like Protein (MAL) *single nucleotide polymorphisms (SNPs), and *TLR2 *length polymorphisms in 170 residents of two regions of Kenya where malaria transmission is stable and high (holoendemic) or episodic and low, 346 residents of a malaria holoendemic region of Papua New Guinea, and 261 residents of North America of self-identified ethnicity.

**Results:**

The difference in historical malaria exposure between the two Kenyan sites has significantly increased the frequency of malaria protective alleles *glucose-6-phoshpate dehydrogenase *(*G6PD*) and *Hemoglobin S (HbS) *in the holoendemic site compared to the episodic transmission site. However, this study detected no such difference in the *TLR2, TLR4, TLR9*, and *MAL *allele frequencies between the two study sites. All polymorphisms were in Hardy Weinberg Equilibrium in the Kenyan and Papua New Guinean populations. *TLR9 *SNPs and length polymorphisms within the *TLR2 *5' untranslated region were the only mutant alleles present at a frequency greater than 10% in all populations.

**Conclusion:**

Similar frequencies of *TLR2, TLR4, TLR9*, and *MAL *genetic polymorphisms in populations with different histories of malaria exposure suggest that these innate immune pathways have not been under strong selective pressure by malaria. Genotype frequencies are consistent with Hardy-Weinberg Equilibrium and the Neutral Theory, suggesting that genetic drift has influenced allele frequencies to a greater extent than selective pressure from malaria or any other infectious agents in these populations.

## Background

Every year 350–500 million cases of malaria occur worldwide, and up to 2.5 million of these individuals are estimated to die, mainly from neurologic complications, severe anaemia or respiratory distress due to *Plasmodium falciparum *[1]. The burden of malaria mortality now and in recent human evolutionary history has been greatest in children under the age of five years, consistent with the notion that malaria has had a powerful effect on selection of the human genome. Indeed, based on observations of the geographic overlap between malaria endemicity and the frequency of erythroid variants such as thalassaemia, Haldane hypothesized in the mid 20^th ^century that such polymorphisms were under selective pressure, because they were protective against severe malaria infection and thereby increased the reproductive fitness of individuals living in malaria endemic areas [2,3].

Toll-like receptors are innate immune receptors that bind to conserved structural motifs expressed by microbial pathogens. TLR2, TLR4, TLR9, and downstream signaling pathways of these proteins have recently been implicated in human malaria pathogenesis [4-6]. TLR2 is expressed on the cell surface, where it is activated by bacterial lipopeptides and fungal and *Mycoplasma *ligands [7-9]. Malaria-derived glycosylphosphatidylinositol (GPI) has been identified as a potential malaria "toxin" by activating TLR2 signaling [5]. The *TLR2 *gene is located on chromosome 4q32 and is comprised of 3 exons, which encode 784 amino acids [10,11]. A 22 bp insertion/deletion polymorphism (*TLR2 *Δ22) in the first untranslated exon was observed to be highly polymorphic in a Japanese population but was not associated with a disease phenotype [12]. A GT dinucleotide that varies by approximately 12 to 30 repeats (GT_n_) is present within the second intron, approximately 100 bp upstream of the translational start site. Repeats of varying length have been associated with susceptibility to tuberculosis, reversal reactions in leprosy, and colorectal cancer [13-15]. Shorter repeats are associated with reduced TLR2 reporter activity and TLR2 surface expression *in vitro *[13]. A non-synonymous SNP in the third exon of *TLR2*, Arg753Gln, is associated with susceptibility to tuberculosis [16], and presumably because of its location in the intracellular domain, abolishes downstream signaling *in vitro *[17]. TLR4 is expressed on the cell surface and is activated by bacterial lipopolysaccharide [18,19]. *Plasmodium falciparum *GPI may weakly activate TLR4 [5]. The gene is on chromosome 9q32-q33, consists of 4 exons, and spans approximately 19 kb [10,20]. Two non-synonymous *TLR4 *SNPs (Asp299Gly and Thr399Ile) are in the extracellular domain, and commonly co-segregate in European Caucasian but not in African populations [21,22]. These SNPs have been associated with various infectious and inflammatory diseases (reviewed in [22]). Ghanaian children with the Asp299Gly and Thr399Ile alleles had an increased risk of severe malaria [4]. These TLR4 mutations confer a hyporesponsive phenotype *in vitro *in some studies, [23] but not in others [22,24]. TLR9 is expressed in endosomal compartments, where it binds to and is activated by bacterial DNA with unmethylated CpG motifs [25,26]. Malaria haemozoin and/or parasite DNA have been identified as potential TLR9 ligands [27,28]. *TLR9 *spans 5 kb on chromosome 3p21.3, consists of 2 exons, and encodes 1028 amino acids [29]. Three *TLR9 *SNPs are present at approximately 15% or greater allele frequency in North American populations [30]. Two of these SNPs, -1486T/C and -1237T/C, are located within the promoter; 1174G/A is in the intron. TLR9 -1237 T/C has been associated with susceptibility to Crohn's Disease, atopic eczema, and asthma [31-33]. Both *TLR4 *Asp299Gly and *TLR9 *-1486T/C were associated with low birth weight and maternal anaemia in a malaria endemic population [34]. MAL (also known as Toll-Interleukin-1 Receptor domain-containing Adaptor Protein, TIRAP) is a downstream adaptor protein common to both the TLR2 and TLR4 signaling pathways [35,36]. The gene spans 14.5 kb on chromosome 11q24.2, consists of 8 exons, and encodes a 221 amino acid protein. Individuals heterozygous for a Ser/Leu mutation at amino acid 180 were reported to be protected from invasive pneumococcal disease, bacteraemia, malaria, and tuberculosis in an analysis of several case-control studies conducted in the UK and sub-Saharan Africa [6]. A subsequent study found protection against tuberculosis in a population of Colombians [37], but no protection was revealed in an analysis comparing tuberculosis cases and controls from Russia, Ghana, and Indonesia [38]. The Ser to Leu amino acid change abolishes the ability of TLR2 to recruit MAL for downstream signaling *in vitro *[6].

The hypothesis of this study was that the above *TLR *and *MAL *genes are under selective pressure by malaria and therefore, that the frequencies of the SNPs observed in these genes would vary significantly among populations historically and currently exposed to differing levels of malaria transmission and morbidity. For example, in the two Kenyan study sites examined here, the frequencies of sickle cell trait and G6PD deficiency are significantly greater in the holoendemic site compared to the episodic transmission site [39], where malaria transmission was introduced during the first half of the 20^th ^century [40,41]. To test this hypothesis, residents living in areas of varying malaria endemicity in sub-Saharan Africa and Papua New Guinea were genotyped for *TLR2, 4, 9*, and *MAL *polymorphisms. Furthermore, in order to put these data in the context of innate immune polymorphisms of populations with no recent history of malaria exposure, North Americans of self-identified ethnicity were also analyzed.

## Methods

### Study populations

*TLR *and *MAL *genetic polymorphisms were evaluated in 170 individuals from Kenya, 346 individuals from Papua New Guinea, and 261 North Americans of self identified ethnicity (total n = 777). Kenyan and Papua New Guinean study population characteristics in terms of age structure and malaria infection prevalence are presented in additional file 1 (table S1). The samples from Kenyans were collected from residents of two villages in the western part of the country, Kanyawegi (n = 91) and Kipsamoite (n = 79), as part of an earlier immunoepidemiologic study [42]. All study participants were asymptomatic with no malaria-related illness when blood for genotyping was obtained. Malaria transmission in the lowland area of Kanyawegi is holoendemic. Annual entomologic inoculation rates may exceed 300 infectious bites per person [43]. Kipsamoite is located in a highland area in the Nandi district of Rift Valley Province where malaria transmission is unstable and characterized by periods with extremely low malaria infection prevalence [44]. During the course of this study, uncomplicated malaria cases peaked in Nandi during the months of May to August, during which time approximately 200 cases were reported by the Kipsamoite Health Center [44]. The 346 Papua New Guinean DNA samples were collected from people of the Urat and Urim linguistic groups in the Dreikikir district of East Sepik Province. These individuals were enrolled in a study examining mass drug administration to eliminate filariasis. During the course of the study, infection rates with various *Plasmodium *spp. and *P. falciparum *asexual densities were obtained. All individuals were asymptomatic with no known episodes of severe malaria morbidity. Malaria transmission is perennial and stable in this area of Papua New Guinea. The entomologic inoculation rate is estimated to be 0.9 infectious bites per person per night, a transmission intensity similar to the holoendemic Kenya site [45]. Mild malaria in this area has a peak prevalence in children less than 5 years old, and severe malaria is most frequently manifested as severe anaemia. DNA from de-identified North American adults was obtained from the National Histocompatibility Laboratory, American Red Cross/University of Maryland Medical System, Baltimore, MD. Race and ethnicity of the North Americans were self-identified. The population was heterogeneous – Asian American (n = 84), Caucasian American of European ancestry (n = 84), and African American (n = 93).

Informed consent, blood sample collection, and genotyping were performed under protocols approved by the Ethical and Institutional Review Boards for Human Investigation at the Kenya Medical Research Institute, the Medical Research Advisory Committee of the Papua New Guinea Department of Health, and University Hospitals of Cleveland/Case Western Reserve University.

### Polymerase chain reaction (PCR)

Genomic DNA was extracted from blood using the QIAamp 96 spin blood kit (QIAGEN, Valencia, CA). PCR was performed using a master mix consisting of 1× PCR buffer, 125 μM dNTPs, 2.5 mM MgCl_2_, 125 nM primers, and 0.8 units *Taq *polymerase in a reaction volume of 25 μl. PCR primers and amplification conditions are listed in additional file 2 (table S2). PCR products for SNP detection were analyzed on a 2% agarose gel prior to LDR-FMA. *TLR2 *Δ 22 genotypes were assigned based on size discrimination of PCR products on a 4% agarose gel. *TLR2 *GT_n _genotypes were assigned based on size discrimination of PCR products run on a 6% polyacrylamide gel as described elsewhere [46]

### Cloning and sequencing

PCR amplification products from local donors were purified using the QIAquick PCR purification kit (QIAGEN, Valencia, CA). Purified PCR products were sent to MWG Biotech, High Point, NC for sequencing. Sequences were analyzed using the Sequencher software (Gene Codes Corporation, Ann Arbor, MI). Sequenced DNA templates were subjected to PCR and used in the LDR-FMA as positive controls.

### SNP genotyping

PCR products were analyzed in a LDR-FMA divided into 3 steps: (1) ligation of oligonucleotides to the SNP, (2) FlexMAP microsphere hybridization, and (3) detection using the Bioplex suspension array system, which includes a fluorescence reader and the Bio-Plex Manager analytical software (Bio-Rad Laboratories, Hercules, CA). This procedure is described in detail elsewhere [47]. Conserved and allele-specific probe sequences are listed in additional file 3 (table S3).

A multiplex assay was used for simultaneous detection of *TLR2*, *TLR9*, and *MAL *SNPs. *TLR4 *SNPs were evaluated in a separate multiplexed LDR. Equal volumes of each PCR product were mixed, and 1 μl was added to the LDR. Conditions for the LDR step of this assay are described elsewhere [47].

### Statistical analysis

Mean Fluorescence Intensity (MFI) values were used to calculate the allelic ratio for each SNP by dividing the allele-specific MFI value by the sum of the MFI values for that SNP (allele A/A+B = A_n_, and allele B/A+B = B_n_), where A and B are the 2 alleles of a SNP. To be homozygous for a particular allele, the ratio must be > 0.75. To be heterozygous, the ratio of the 2 alleles must be between 0.25 and 0.75. Consequently, an allele included in a ratio of < 0.25 is considered not present. Normalized values for A_n _and B_n _were divided, and the quotient was log-transformed. The mean and 95% confidence interval (CI) of the log-transformed quotients for the 777 individuals genotyped in this study are presented in additional file 4 (table S4).

Allele (or haplotype) and genotype (diplotype) frequencies were calculated for the different polymorphisms, the Hardy-Weinberg exact test (estimation of p-values by the Markov chain method), and the Ewens-Watterson homozygosity test was performed for each population using Arlequin version 3.01 . Differences in allele frequencies among study groups were determined by chi-square analysis using SPSS for Windows (version 13.0). A p-value < 0.05 was considered to be significant. Statistical comparisons of allele frequencies were made between the following groups; Kenya malaria holoendemic vs. Kenya episodic transmission: each of the Kenyan study populations vs. African American, Kenya holoendemic vs. Papua New Guinea holoendemic, and Papua New Guinea holoendemic vs. Asian Americans. Linkage disequilibrium analysis was performed using SHEsis [48].

## Results

Table 1 describes the mutant allele frequencies for the *TLR2*, *TLR4*, *TLR9*, and *MAL *SNPs, *TLR2 *Δ22, and *TLR2 *GT_n _polymorphisms in the populations from Kenya, Papua New Guinea, and North America.

**Table 1:** *TLR2, 4, 9*, and *MAL *mutant allele frequencies in various populations

		PNG	Kenya		North America		
Gene	Mutant allele	lowlands	lowlands	Highlands	African	Caucasian	Asian
TLR2							
	Δ22	0.131^I^	0.298	0.326^II^	0.215	0.131	0.256^VI^
	GTS	0.091	0.082	0.145	0.111	0.036	0.212
	GTM	0.522^I^	0.799	0.761	0.721	0.583	0.394^VI^
	GTL	0.387	0.120	0.094	0.168	0.381	0.394
	Arg753Gln	0.000^V^	0.000	0.000	0.011	0.038	0.000
TLR4							
	Asp299Gly	0.000^V^	0.071^III^	0.054^II^	0.131	0.086	0.042
	Thr399Ile	0.000^V^	0.015	0.007	0.028	0.095	0.036
TLR9							
	-1486T/C	0.371^I^	0.270	0.25^IV^	0.343	0.340	0.361
	-1237T/C	0.000	0.351	0.362	0.337	0.130	0.066
	1174G/A	0.624^I^	0.403	0.481^II^	0.341	0.525	0.590
MAL							
	Ser180Leu	0.149	0.000	0.025	0.056	0.160	0.060^VI^

GTS < 16 repeats, GTM 16–23 repeats, GTL > 23 repeats.

^I^p < 0.05, compared with Kenyan lowlands

^II^p < 0.05 compared to African Americans

^III^p = 0.062 compared to African Americans

^IV^p = 0.067 compared to African Americans

^V^tested previously [50]

^VI^p < .005 compared to PNG

### *TLR2 *polymorphisms

The *TLR2 *Δ22 polymorphism in the 5' untranslated region was present in all populations examined at an allele frequency >10%. The lowest value was in Papua New Guineans (13.1%), compared with 32.6% for the Kenyan episodic transmission site. Although the holoendemic Kenyan and Papua New Guinea sites have similar malaria transmission intensities, this allele was significantly less common in Papua New Guinea compared to the Kenya holoendemic site (p < 0.001). Allele frequencies were similar for Kenyan study participants from the holoendemic and episodic transmission sites.

**Figure 1:**
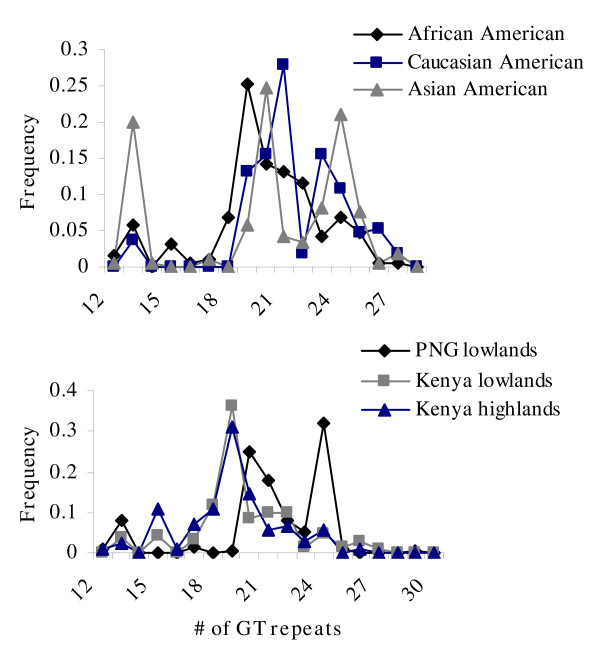
**Frequency of GT repeat polymorphism (GTn) among residents of malaria endemic and malaria non-endemic areas**.

The GT_n _repeat in the 5' untranslated region of *TLR2 *was highly polymorphic in all populations examined. Consistent with how this genetic variation has been analyzed previously [13,49], the alleles were classified as short (<16), medium (16–23), and long (>23) repeats. The distribution of the length of the repeat is displayed in Figure 1, and frequencies of the allele are presented in Table 1. The medium length allele accounted for >50% of total alleles in all populations with the exception of Asian Americans, where it was 39.4%.

A previous report showed that the *TLR2 *SNP Arg753Gln is absent in Urat and Urim speakers from Papua New Guinea [50]. Therefore, this SNP was not tested here. Arg753Gln was also absent in Kenyans but present in North Americans, consistent with previous reports [51].

### *TLR4*, *TLR9*, and *MAL *SNP allele frequencies

The two *TLR4 *SNPs examined in this study are absent in the Urat and Urim linguistic groups in Papua New Guinea [50] and therefore, were not tested here. The predicted Asp299Gly and Thr399Ile amino acid mutations were present at frequencies that were not statistically different for the Kenyans residing in the malaria holoendemic and episodic transmission areas (range 0.7–7.1%). With the exception of -1237C in Papua New Guineans and Asian Americans, all *TLR9 *SNPs were present at a frequency greater than 10%. Both -1486T/C and 1174G/A were more common in Papua New Guinea compared to the Kenya holoendemic site (p = 0.026 and p < 0.001, respectively). There were no significant differences in *TLR9 *SNP frequencies associated with different malaria exposures in Kenya. The *MAL *180 mutant allele was most common in Papua New Guineans (14.9%). In contrast, this allele was completely absent in the holoendemic Kenyan transmission site, and present in only 2.5% in the episodic transmission site.

### Frequencies of *TLR2*, *TLR 4*, *TLR9*, and *MAL *genotypes

Table 2 shows the genotype frequencies for *TLR2 *Δ22, *TLR4*, *TLR9*, and the *MAL *polymorphisms. All three *TLR2 *Δ22 genotypes were found in all populations, and were in Hardy-Weinberg equilibrium. The *TLR4, TLR9*, and *MAL *SNPs were in Hardy-Weinberg equilibrium in both Kenya and Papua New Guinea. In contrast to Papua New Guineans where -1237C was absent, this allele was present in Asian Americans, for whom there were fewer heterozygotes than expected (p = 0.022). The *MAL *genotypes were in Hardy-Weinberg equilibrium in all populations except in African Americans, where there was a deficit of heterozygosity (p = 0.02).

**Table 2:** *TLR2, 4, 9*, and *MAL *genotypes in various populations

		PNG	Kenya		North America		
Polymorphism^I^	genotype	lowlands	lowlands	highlands	African	Caucasian	Asian
TLR2 Δ22	WW	253(76)	41(49)	33(48)	58(62)	64(76)	49(58)
	WD^II^	73(22)	36(43)	27(39)	30(32)	18(21)	27(32)
	DD	7(2)	7(8)	9(13)	5(5)	2(2)	8(10)
TLR4 Asp299Gly	Asp/Asp	906(100)^IV^	78(86)	66(89)	65(74)	67(83)	76(92)
	Asp/Gly	0	13(14)	8(11)	23(26)	14(17)	7(8)
TLR4 Thr399Ile	Thr/Thr	906(100)^IV^	64(97)	69(99)	84(94)	64(81)	77(93)
	Thr/Ile	0	2(3)	1(1)	5(6)	15(19)	6(7)
TLR9 -1486	TT	137(41)	47(54)	41(54)	37(42)	36(44)	34(41)
	CT	146(44)	33(38)	32(42)	43(48)	35(43)	38(46)
	CC	51(15)	7(8)	3(4)	9(10)	10(12)	11(13)
TLR9 -1237	TT	334(100)	39(45)	29(38)	36(40)	61(75)	74(89)
	CT	0	35(40)	39(51)	46(52)	19(23)	7(8)^III^
	CC	0	13(15)	8(11)	7(8)	1(1)	2(2)
TLR9 1174	GG	53(16)	29(32)	17(22)	35(43)	22(27)	14(17)
	GA	147(44)	47(53)	47(60)	38(46)	33(41)	40(48)
	AA	136(41)	12(14)	14(18)	9(11)	26(32)	29(35)
MAL 180	Ser/Ser	254(73)	89(100)	75(95)	82(91)	53(68)	73(88)
	Ser/Leu	81(23)	0	4(5)	6(7)^III^	25(32)	10(12)
	Leu/Leu	11(3)	0	0	2(2)	0	0

^I^no.(%)

^II^D is deletion allele, W is wild type/insertion allele

^III^p < .05, Hardy-Weinberg test of equilibrium, deficit of heterozygosity

^IV^genotyped previously [50]

### Inferred haplotypes and linkage disequilibrium

Statistically inferred haplotypes for *TLR2, TLR4*, and *TLR9 *are described in Table 3. Of six possible *TLR2 *haplotype combinations, all were present in Papua New Guineans and North Americans. The deletion allele did not co-segregate with any of the GT repeat length alleles, as determined in linkage disequilibrium analysis where two polymorphisms were considered in linkage disequilibrium if the values for D' and r^2 ^were >0.9 (data not shown). The Asp299Gly and Thr399Ile SNPs in the *TLR4 *gene commonly co-segregated in Caucasian Americans. Consistent with these data, the two polymorphisms were in linkage disequilibrium as measured by D' and r^2 ^(data not shown). In Kenya, the *TLR4 *mutations co-segregated less frequently, and the Gly-Thr haplotype was present at 5–6% frequency in both the episodic transmission and holoendemic study sites. This haplotype was also found in African Americans and Asian Americans. There was no linkage disequilibrium between Asp299Gly and Thr399Ile in the Kenyans, African Americans, or Asians Americans (data not shown). The most common *TLR9 *haplotypes were CTG and TTA, except in Kenyans and African Americans, where TCG and TTA were the most common. There were fewer *TLR9 *haplotypes present in Papua New Guineans compared to the other populations, largely due to the absence of the -1237 allele. There was linkage disequilibrium between all *TLR9 *SNP pairs present in Papua New Guineans (data not shown). This linkage disequilibrium was absent in the Kenyan and Asian American populations.

**Table 3:** *TLR2, TLR4*, and *TLR9 *haplotypes in various populations

		PNG	Kenya		North America		
Gene	Haplotype	lowlands	lowlands	highlands	African	Caucasian	Asian
TLR2^I^							
	DL	0.029	0.000	0.000	0.000	0.000	0.086
	DM	0.011	0.258	0.239	0.164	0.099	0.105
	DS	0.093	0.041	0.092	0.051	0.033	0.063
	WL	0.355	0.110	0.100	0.161	0.388	0.300
	WM	0.509	0.559	0.515	0.572	0.480	0.293
	WS	0.003	0.033	0.054	0.052	0.000	0.154
TLR4^II^							
	Asp-Thr	1^IV^	0.924	0.943	0.869	0.905	0.952
	Gly-Ile	0	0.015	0.007	0.028	0.089	0.030
	Gly-Thr	0	0.061	0.050	0.102	0.000	0.012
	Asp-Ile	0	0.000	0.000	0.000	0.006	0.006
TLR9^III^							
	C C A	0.000	0.006	0.007	0.000	0.000	0.000
	C C G	0.000	0.075	0.099	0.092	0.000	0.011
	C T A	0.003	0.000	0.011	0.018	0.000	0.007
	C T G	0.368	0.205	0.136	0.244	0.353	0.343
	T C G	0.000	0.266	0.254	0.256	0.115	0.055
	T T A	0.625	0.396	0.455	0.324	0.526	0.584
	T T G	0.005	0.051	0.037	0.066	0.006	0.000

^I^Δ22-GT_n _haplotypes

D is deletion allele, W is wild type

S < 16 repeats, M 16–23 repeats, L > 23 repeats.

^II^First amino acid is 299, second amino acid is 399

^III^First nucleotide is -1486, second is -1237, and third is 1174.

^IV^Genotyped previously [50]

## Discussion

There is compelling evidence that evolution of the human genome has involved selective pressure exerted by microbial pathogens. Perhaps one of the best characterized examples of this natural selection process is historical exposure to falciparum malaria and its' association with the β-globin SNP underlying the haemoglobin S sickle cell trait. The frequency of this mutant allele (and other β-globin variants such as haemoglobin C) is greater than 25% in populations in Sub-Saharan Africa where it has been observed to mediate a strong protective effect against severe malaria [52]. Data reported here provide information relevant to the growing interest in understanding how innate immunity may contribute to malaria susceptibility. Information on the genetics of innate immunity should help define the basis of individual and population variation in susceptibility to malaria pathogenesis and may facilitate efforts to develop vaccines, vaccine adjuvants, and treatments to fight this infectious disease.

This study focused on *TLR2*, *TLR4*, *TLR9 *and *MAL *because these genes have been implicated in human malaria pathogenesis. Evidence for this includes *in vitro *experiments in which engagement of the immune receptor by putative malaria toxins and activation of downstream signaling pathways lead to production of pro-inflammatory cytokines [5,27,28], and from case-control studies in which the frequencies of selected polymorphisms among individuals with severe malaria illness are compared with those of ethnically and age-matched residents with asymptomatic malaria [4,6]. The results presented here suggest that none of the *TLR *or *MAL *polymorphisms examined have been under strong selective pressure by malaria. Although the protective haemoglobin S and G6PD polymorphisms are significantly more common in Kenyan residents of the malaria holoendemic area compared to residents of the nearby episodic low transmission area [39], statistically significant differences in mutant allele frequencies of the *TLR *or *MAL *polymorphisms were not detected. A notable finding in the Papua New Guinea population is concerned with the *MAL *SNP that confers a Ser→Leu amino acid change. The mutation abrogates downstream signaling following ligand engagement of TLR2 and TLR4. The frequency of the mutant allele in the Papua New Guinea population was high (14.9%, see Table 1) compared to the other populations. Moreover, identification of homozygous individuals for the mutant Leu allele (11 of 254 individuals, see Table 2) is of potential interest since previous studies have suggested that heterozygosity affords protection against not only malaria but also bacterial infections. Homozygosity for this allele has not been observed in African populations (e.g. Khor, et al [6] reported no homozygous individuals among the 4902 West and East Africans genotyped in their study.) Third, all of the polymorphisms in malaria endemic populations studied here were in Hardy-Weinberg equilibrium, suggesting that selective pressure by malaria (or other population-wide effects such as bacterial infection, starvation, population size, non-random mating, etc), has not affected the frequency and distribution of the allele. Additionally, these alleles followed the Neutral Theory as determined by a p-value < 0.05 for the Ewens-Watterson test of homozygosity. For the polymorphisms examined in these study populations, the frequency of observed homozygotes was not significantly different than the homozygote frequency expected under the Neutral Theory. This suggests that genetic drift rather than natural selection, by malaria or any other agent, has been more significant in determining these allele frequencies.

The lack of difference in the frequency of these polymorphisms among populations with varying levels of malaria endemicity may be because these innate immune pathways truly do not alter susceptibility to severe malaria. However, it is important to recognize alternative explanations. The number of individuals sampled may be insufficient to detect statistically small but biologically meaningful differences. Whereas *MAL *Ser180Leu and the *TLR4 *SNPs in other populations have respectively, been associated with protection and susceptibility to malaria, these associations may not exist in the Kenyan or Papua New Guinean populations examined here. For example, HLA-B*5301 and HLA-DRB1*1302 were associated with resistance to severe malaria in Gambia [53], whereas this association was not found in Kenya [54]. *TNF *and *ICAM-1 *polymorphisms and their association with malaria susceptibility has also been inconsistent across various populations (reviewed in [52]). Other counter-selective forces in these populations also may be acting to limit the spread of the polymorphisms in the genes studied here. Polymorphisms that are protective against malaria may increase the risk of non-malarial infectious diseases. For example, recently it was demonstrated that Duffy blood group-negativity, which confers complete protection from blood stage *P. vivax *in Africans [55], increases the risk of HIV infection in African Americans and may also contribute to the burden of HIV infection in Africa [56]. Because TLRs and their downstream adaptor proteins are critical in the responses to different pathogens, it is likely that similarly complex selective pressures act on these genes.

This study is the first to describe the *TLR*2 5' untranslated polymorphisms in Kenyans and Papua New Guineans from malaria endemic areas. These polymorphisms presumably affect transcription and/or translation. The Δ22 polymorphism is located in the first untranslated exon of *TLR2*, approximately 60 bp downstream of an NF-kB site critical for chromatin remodeling and binding of transcription factors [57]. The *in vitro *phenotypes of Δ22 and GT_n _need to be investigated more thoroughly to determine their effect on mRNA transcription and/or translation. Although these polymorphisms have not been studied in malaria case-control studies, TLR2 is potentially involved in the pathogenesis of malaria because of its putative role as a receptor for *P. falciparum*-derived GPI [5].

Future studies with larger sample sizes, additional SNPs, and other genetic loci are needed to determine more completely whether falciparum malaria has been involved in natural selection of genes that regulate innate immunity in humans. Advances in analytic and molecular tools such as tagged SNPs, such as those now available in the International HapMap project, also make it possible to identify signatures of natural selection in these and other genetic loci without *a priori *knowledge of their biologic function and contribution to malaria pathogenesis [58].

## Conclusion

The goal of this study was to identify *TLR *and *TLR *adaptor polymorphisms under selective pressure by malaria. The data suggest that history of malaria exposure of the two Kenyan populations has not been involved in selection of *TLR2, 4*, and *9 *or *MAL *polymorphisms. Case-control studies comparing the frequencies of these polymorphisms among individuals with severe malaria with those with asymptomatic malaria infection need to be conducted to define more completely the potential role of these innate immune genes in human malaria pathogenesis.

## Competing interests

The authors declare that they have no competing interests.

## Authors' contributions

JG performed and analyzed genotyping assays and drafted manuscript. JK conceived of the study, participated in its design and helped draft the manuscript. PZ participated in the design of the genotyping strategy and assisted in the analysis of the genotyping data. AM, JV, and MB were involved in collection of samples and the design of population-based field studies and helped write the manuscript.

## Supplementary Material

Additional file 1 **Malariometric characteristics of Kenyan and Papua New Guinean study subjects.** This file contains age, sex, and parasitaemia information for the Kenyan and Papua New Guinean study participants. Click here for file

Additional file 2 **PCR primers and amplification conditions.** This file contains sequences for the PCR primers used and amplification conditions. Click here for file

Additional file 3 **LDR-FMA primers for SNP genotyping.** This file contains the oligonucleotide sequences for the LDR-FMA reaction, as well as the SNPs examined and their accession number. Click here for file

Additional file 4 **Mean and 95% confidence interval (CI) values of allelic ratios to determine genotypes.** This file contains the mean and 95% confidence intervals of the MFI values from the LDR-FMA reaction used to determine genotypes. Click here for file

## References

[B1] Newton CR, Hien TT, White N (2000). Cerebral malaria. J Neurol Neurosurg Psychiatry.

[B2] Haldane J (1949). The Rate of Mutations of Human Genes. Proceedings of the Eighth International Congress on Genetics Sweden: Hereditas.

[B3] Haldane J (1949). Disease and evolution. Ric Sci.

[B4] Mockenhaupt FP, Cramer JP, Hamann L, Stegemann MS, Eckert J, Oh NR, Otchwemah RN, Dietz E, Ehrhardt S, Schroder NW, Bienzle U, Schumann RR (2006). Toll-like receptor (TLR) polymorphisms in African children: Common TLR-4 variants predispose to severe malaria. Proc Natl Acad Sci USA.

[B5] Krishnegowda G, Hajjar AM, Zhu J, Douglass EJ, Uematsu S, Akira S, Woods AS, Gowda DC (2005). Induction of proinflammatory responses in macrophages by the glycosylphosphatidylinositols of Plasmodium falciparum: cell signaling receptors, glycosylphosphatidylinositol (GPI) structural requirement, and regulation of GPI activity. J Biol Chem.

[B6] Khor CC, Chapman SJ, Vannberg FO, Dunne A, Murphy C, Ling EY, Frodsham AJ, Walley AJ, Kyrieleis O, Khan A, Aucan C, Segal S, Moore CE, Knox K, Campbell SJ, Lienhardt C, Scott A, Aaby P, Sow OY, Grignani RT, Sillah J, Sirugo G, Peshu N, Williams TN, Maitland K, Davies RJ, Kwiatkowski DP, Day NP, Yala D, Crook DW, Marsh K, Berkley JA, O'Neill LA, Hill AV (2007). A Mal functional variant is associated with protection against invasive pneumococcal disease, bacteremia, malaria and tuberculosis. Nat Genet.

[B7] Ozinsky A, Underhill DM, Fontenot JD, Hajjar AM, Smith KD, Wilson CB, Schroeder L, Aderem A (2000). The repertoire for pattern recognition of pathogens by the innate immune system is defined by cooperation between toll-like receptors. Proc Natl Acad Sci USA.

[B8] Lien E, Sellati TJ, Yoshimura A, Flo TH, Rawadi G, Finberg RW, Carroll JD, Espevik T, Ingalls RR, Radolf JD, Golenbock DT (1999). Toll-like receptor 2 functions as a pattern recognition receptor for diverse bacterial products. J Biol Chem.

[B9] Takeuchi O, Kaufmann A, Grote K, Kawai T, Hoshino K, Morr M, Muhlradt PF, Akira S (2000). Cutting edge: preferentially the R-stereoisomer of the mycoplasmal lipopeptide macrophage-activating lipopeptide-2 activates immune cells through a toll-like receptor 2- and MyD88-dependent signaling pathway. J Immunol.

[B10] Rock FL, Hardiman G, Timans JC, Kastelein RA, Bazan JF (1998). A family of human receptors structurally related to Drosophila Toll. Proc Natl Acad Sci USA.

[B11] Haehnel V, Schwarzfischer L, Fenton MJ, Rehli M (2002). Transcriptional regulation of the human toll-like receptor 2 gene in monocytes and macrophages. J Immunol.

[B12] Noguchi E, Nishimura F, Fukai H, Kim J, Ichikawa K, Shibasaki M, Arinami T (2004). An association study of asthma and total serum immunoglobin E levels for Toll-like receptor polymorphisms in a Japanese population. Clin Exp Allergy.

[B13] Yim JJ, Lee HW, Lee HS, Kim YW, Han SK, Shim YS, Holland SM (2006). The association between microsatellite polymorphisms in intron II of the human Toll-like receptor 2 gene and tuberculosis among Koreans. Genes Immun.

[B14] Boraska Jelavic T, Barisic M, Drmic Hofman I, Boraska V, Vrdoljak E, Peruzovic M, Hozo I, Puljiz Z, Terzic J (2006). Microsatelite GT polymorphism in the toll-like receptor 2 is associated with colorectal cancer. Clin Genet.

[B15] Bochud PY, Hawn TR, Siddiqui MR, Saunderson P, Britton S, Abraham I, Argaw AT, Janer M, Zhao LP, Kaplan G, Aderem A (2008). Toll-like receptor 2 (TLR2) polymorphisms are associated with reversal reaction in leprosy. J Infect Dis.

[B16] Ogus AC, Yoldas B, Ozdemir T, Uguz A, Olcen S, Keser I, Coskun M, Cilli A, Yegin O (2004). The Arg753GLn polymorphism of the human toll-like receptor 2 gene in tuberculosis disease. Eur Respir J.

[B17] Schroder NW, Diterich I, Zinke A, Eckert J, Draing C, von Baehr V, Hassler D, Priem S, Hahn K, Michelsen KS, Hartung T, Burmester GR, Gobel UB, Hermann C, Schumann RR (2005). Heterozygous Arg753Gln polymorphism of human TLR-2 impairs immune activation by Borrelia burgdorferi and protects from late stage Lyme disease. J Immunol.

[B18] Chow JC, Young DW, Golenbock DT, Christ WJ, Gusovsky F (1999). Toll-like receptor-4 mediates lipopolysaccharide-induced signal transduction. J Biol Chem.

[B19] Hoshino K, Takeuchi O, Kawai T, Sanjo H, Ogawa T, Takeda Y, Takeda K, Akira S (1999). Cutting edge: Toll-like receptor 4 (TLR4)-deficient mice are hyporesponsive to lipopolysaccharide: evidence for TLR4 as the Lps gene product. J Immunol.

[B20] Rehli M, Poltorak A, Schwarzfischer L, Krause SW, Andreesen R, Beutler B (2000). PU.1 and interferon consensus sequence-binding protein regulate the myeloid expression of the human Toll-like receptor 4 gene. J Biol Chem.

[B21] Ferwerda B, McCall MB, Alonso S, Giamarellos-Bourboulis EJ, Mouktaroudi M, Izagirre N, Syafruddin D, Kibiki G, Cristea T, Hijmans A, Hamann L, Israel S, ElGhazali G, Troye-Blomberg M, Kumpf O, Maiga B, Dolo A, Doumbo O, Hermsen CC, Stalenhoef AF, van Crevel R, Brunner HG, Oh DY, Schumann RR, de la Rua C, Sauerwein R, Kullberg BJ, Ven AJ van der, Meer JW van der, Netea MG (2007). TLR4 polymorphisms, infectious diseases, and evolutionary pressure during migration of modern humans. Proc Natl Acad Sci USA.

[B22] Misch EA, Hawn TR (2008). Toll-like receptor polymorphisms and susceptibility to human disease. Clin Sci (Lond).

[B23] Arbour NC, Lorenz E, Schutte BC, Zabner J, Kline JN, Jones M, Frees K, Watt JL, Schwartz DA (2000). TLR4 mutations are associated with endotoxin hyporesponsiveness in humans. Nat Genet.

[B24] Erridge C, Stewart J, Poxton IR (2003). Monocytes heterozygous for the Asp299Gly and Thr399Ile mutations in the Toll-like receptor 4 gene show no deficit in lipopolysaccharide signalling. J Exp Med.

[B25] Latz E, Schoenemeyer A, Visintin A, Fitzgerald KA, Monks BG, Knetter CF, Lien E, Nilsen NJ, Espevik T, Golenbock DT (2004). TLR9 signals after translocating from the ER to CpG DNA in the lysosome. Nat Immunol.

[B26] Hemmi H, Takeuchi O, Kawai T, Kaisho T, Sato S, Sanjo H, Matsumoto M, Hoshino K, Wagner H, Takeda K, Akira S (2000). A Toll-like receptor recognizes bacterial DNA. Nature.

[B27] Parroche P, Lauw FN, Goutagny N, Latz E, Monks BG, Visintin A, Halmen KA, Lamphier M, Olivier M, Bartholomeu DC, Gazzinelli RT, Golenbock DT (2007). Malaria hemozoin is immunologically inert but radically enhances innate responses by presenting malaria DNA to Toll-like receptor 9. Proc Natl Acad Sci USA.

[B28] Coban C, Ishii KJ, Kawai T, Hemmi H, Sato S, Uematsu S, Yamamoto M, Takeuchi O, Itagaki S, Kumar N, Horii T, Akira S (2005). Toll-like receptor 9 mediates innate immune activation by the malaria pigment hemozoin. J Exp Med.

[B29] Du X, Poltorak A, Wei Y, Beutler B (2000). Three novel mammalian toll-like receptors: gene structure, expression, and evolution. Eur Cytokine Netw.

[B30] Lazarus R, Klimecki WT, Raby BA, Vercelli D, Palmer LJ, Kwiatkowski DJ, Silverman EK, Martinez F, Weiss ST (2003). Single-nucleotide polymorphisms in the Toll-like receptor 9 gene (TLR9): frequencies, pairwise linkage disequilibrium, and haplotypes in three U.S. ethnic groups and exploratory case-control disease association studies. Genomics.

[B31] Torok HP, Glas J, Tonenchi L, Bruennler G, Folwaczny M, Folwaczny C (2004). Crohn's disease is associated with a toll-like receptor-9 polymorphism. Gastroenterology.

[B32] Novak N, Yu CF, Bussmann C, Maintz L, Peng WM, Hart J, Hagemann T, Diaz-Lacava A, Baurecht HJ, Klopp N, Wagenpfeil S, Behrendt H, Bieber T, Ring J, Illig T, Weidinger S (2007). Putative association of a TLR9 promoter polymorphism with atopic eczema. Allergy.

[B33] Lachheb J, Dhifallah IB, Chelbi H, Hamzaoui K, Hamzaoui A (2008). Toll-like receptors and CD14 genes polymorphisms and susceptibility to asthma in Tunisian children. Tissue Antigens.

[B34] Mockenhaupt FP, Hamann L, von Gaertner C, Bedu-Addo G, von Kleinsorgen C, Schumann RR, Bienzle U (2006). Common polymorphisms of toll-like receptors 4 and 9 are associated with the clinical manifestation of malaria during pregnancy. J Infect Dis.

[B35] Fitzgerald KA, Palsson-McDermott EM, Bowie AG, Jefferies CA, Mansell AS, Brady G, Brint E, Dunne A, Gray P, Harte MT, McMurray D, Smith DE, Sims JE, Bird TA, O'Neill LA (2001). Mal (MyD88-adapter-like) is required for Toll-like receptor-4 signal transduction. Nature.

[B36] Yamamoto M, Sato S, Hemmi H, Sanjo H, Uematsu S, Kaisho T, Hoshino K, Takeuchi O, Kobayashi M, Fujita T, Takeda K, Akira S (2002). Essential role for TIRAP in activation of the signalling cascade shared by TLR2 and TLR4. Nature.

[B37] Castiblanco J, Varela DC, Castano-Rodriguez N, Rojas-Villarraga A, Hincapie ME, Anaya JM (2008). TIRAP (MAL) S180L polymorphism is a common protective factor against developing tuberculosis and systemic lupus erythematosus. Infect Genet Evol.

[B38] Nejentsev S, Thye T, Szeszko JS, Stevens H, Balabanova Y, Chinbuah AM, Hibberd M, Vosse E van de, Alisjahbana B, van Crevel R, Ottenhoff TH, Png E, Drobniewski F, Todd JA, Seielstad M, Horstmann RD (2008). Analysis of association of the TIRAP (MAL) S180L variant and tuberculosis in three populations. Nat Genet.

[B39] Moormann AM, Embury PE, Opondo J, Sumba OP, Ouma JH, Kazura JW, John CC (2003). Frequencies of sickle cell trait and glucose-6-phosphate dehydrogenase deficiency differ in highland and nearby lowland malaria-endemic areas of Kenya. Trans R Soc Trop Med Hyg.

[B40] Hay SI, Noor AM, Simba M, Busolo M, Guyatt HL, Ochola SA, Snow RW (2002). Clinical epidemiology of malaria in the highlands of western Kenya. Emerg Infect Dis.

[B41] Malakooti MA, Biomndo K, Shanks GD (1998). Reemergence of epidemic malaria in the highlands of western Kenya. Emerg Infect Dis.

[B42] John CC, Moormann AM, Sumba PO, Ofulla AV, Pregibon DC, Kazura JW (2004). Gamma interferon responses to Plasmodium falciparum liver-stage antigen 1 and thrombospondin-related adhesive protein and their relationship to age, transmission intensity, and protection against malaria. Infect Immun.

[B43] Beier JC, Oster CN, Onyango FK, Bales JD, Sherwood JA, Perkins PV, Chumo DK, Koech DV, Whitmire RE, Roberts CR (1994). Plasmodium falciparum incidence relative to entomologic inoculation rates at a site proposed for testing malaria vaccines in western Kenya. Am J Trop Med Hyg.

[B44] John CC, McHugh MM, Moormann AM, Sumba PO, Ofulla AV (2005). Low prevalence of Plasmodium falciparum infection among asymptomatic individuals in a highland area of Kenya. Trans R Soc Trop Med Hyg.

[B45] Bockarie MJ, Alexander N, Bockarie F, Ibam E, Barnish G, Alpers M (1996). The late biting habit of parous Anopheles mosquitoes and pre-bedtime exposure of humans to infective female mosquitoes. Trans R Soc Trop Med Hyg.

[B46] DaRe JT, Mehlotra RK, Michon P, Mueller I, Reeder J, Sharma YD, Stoneking M, Zimmerman PA (2007). Microsatellite polymorphism within pfcrt provides evidence of continuing evolution of chloroquine-resistant alleles in Papua New Guinea. Malar J.

[B47] Mehlotra RK, Ziats MN, Bockarie MJ, Zimmerman PA (2006). Prevalence of CYP2B6 alleles in malaria-endemic populations of West Africa and Papua New Guinea. Eur J Clin Pharmacol.

[B48] Shi YY, He L (2005). SHEsis, a powerful software platform for analyses of linkage disequilibrium, haplotype construction, and genetic association at polymorphism loci. Cell Res.

[B49] Yim JJ, Ding L, Schaffer AA, Park GY, Shim YS, Holland SM (2004). A microsatellite polymorphism in intron 2 of human Toll-like receptor 2 gene: functional implications and racial differences. FEMS Immunol Med Microbiol.

[B50] Hise AG, Hazlett FE, Bockarie MJ, Zimmerman PA, Tisch DJ, Kazura JW (2003). Polymorphisms of innate immunity genes and susceptibility to lymphatic filariasis. Genes Immun.

[B51] Schroder NW, Hermann C, Hamann L, Gobel UB, Hartung T, Schumann RR (2003). High frequency of polymorphism Arg753Gln of the Toll-like receptor-2 gene detected by a novel allele-specific PCR. J Mol Med.

[B52] Kwiatkowski DP (2005). How malaria has affected the human genome and what human genetics can teach us about malaria. Am J Hum Genet.

[B53] Hill AV, Allsopp CE, Kwiatkowski D, Anstey NM, Twumasi P, Rowe PA, Bennett S, Brewster D, McMichael AJ, Greenwood BM (1991). Common west African HLA antigens are associated with protection from severe malaria. Nature.

[B54] Hill AV, Yates SN, Allsopp CE, Gupta S, Gilbert SC, Lalvani A, Aidoo M, Davenport M, Plebanski M (1994). Human leukocyte antigens and natural selection by malaria. Philos Trans R Soc Lond B Biol Sci.

[B55] Tournamille C, Colin Y, Cartron JP, Le Van Kim C (1995). Disruption of a GATA motif in the Duffy gene promoter abolishes erythroid gene expression in Duffy-negative individuals. Nat Genet.

[B56] He W, Neil S, Kulkarni H, Wright E, Agan BK, Marconi VC, Dolan MJ, Weiss RA, Ahuja SK (2008). Duffy antigen receptor for chemokines mediates trans-infection of HIV-1 from red blood cells to target cells and affects HIV-AIDS susceptibility. Cell Host Microbe.

[B57] Johnson CM, Tapping RI (2007). Microbial products stimulate human Toll-like receptor 2 expression through histone modification surrounding a proximal NF-kappaB-binding site. J Biol Chem.

[B58] Walsh EC, Sabeti P, Hutcheson HB, Fry B, Schaffner SF, de Bakker PI, Varilly P, Palma AA, Roy J, Cooper R, Winkler C, Zeng Y, de The G, Lander ES, O'Brien S, Altshuler D (2006). Searching for signals of evolutionary selection in 168 genes related to immune function. Hum Genet.

